# Tryptophan‐sensing receptor GPR142 expression levels are directly regulated by proinflammatory cytokines in ghrelin‐producing cells

**DOI:** 10.1002/2211-5463.13973

**Published:** 2025-01-30

**Authors:** Yoko Ueda, Hiroshi Iwakura, Takuya Ensho, Mika Bando‐Shimizu, Asako Doi, Norihiko Matsutani, Shuhei Morita, Hidefumi Inaba, Hiroyuki Ariyasu, Naoki Fukuda, Keiji Hayata, Toshiyasu Ojima, Masahiro Nishi, Taka‐aki Matsuoka, Hiroki Yamaue, Takashi Akamizu

**Affiliations:** ^1^ Department of Pharmacotherapeutics School of Pharmaceutical Science, Wakayama Medical University Wakayama Japan; ^2^ The First Department of Medicine Wakayama Medical University Wakayama Japan; ^3^ Second Department of Surgery Wakayama Medical University Wakayama Japan; ^4^ Department of Medical Technology, Faculty of Health Sciences Kansai University of Medical Sciences Osaka Japan

**Keywords:** ghrelin, GPR142, proinflammatory cytokines, tryptophan

## Abstract

GPR142 is a tryptophan‐sensing receptor that has been implicated in the regulation of inflammation. In this study, we investigated the relationships between inflammatory cytokine and GPR142 expression by using cellular, animal models, and human stomach samples. We found that addition of TNF‐α, IL‐6, and IL‐1β into the culture of ghrelin‐producing cell line, MGN3‐1 cells, increased GPR142 mRNA expression levels. Lipopolysaccharide (LPS) injection to mice significantly increased GPR142 expression in the stomach, confirming the results observed in the cellular model. GPR142 mRNA expression levels in the stomach samples of morbidly obese patients were positively correlated with TNF‐α, IL‐6, and IL‐1β mRNA levels. Taken together our results suggest that GPR142 expression is under the direct control of proinflammatory cytokines and support further investigation of GPR142 potential roles in inflammation.

## Graphical abstract

GPR142, a tryptophan‐sensing receptor, stimulates gastrointestinal hormones like ghrelin and is linked to inflammation regulation. Proinflammatory cytokines suppress ghrelin production, while tryptophan levels decrease via IDO1 activation. Our findings reveal that proinflammatory cytokines directly regulate GPR142 expression in ghrelin‐producing cells, highlighting its potential role in inflammation and warranting further investigation.

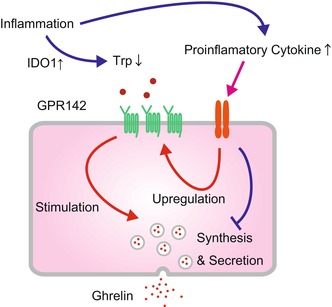

**Abbreviations**
Alb: albumin
ALT: alanine aminotransferase
ANOVA: analysis of variance
AST: aspartate aminotransferase
CAIA: collagen antibody‐induced arthritis
CCK: cholecystokinin
CPR: C‐peptide
Cre: creatinine
CRP: C‐reactive protein
eGFR: estimated glomerular filtration rate
FPKM: fragments per kilobase of exon per million reads mapped
GIP: glucose‐dependent insulinotropic polypeptide
GLP‐1: glucagon‐like peptide‐1
HbA1c: hemoglobin A1c
HDL‐C: high‐density lipoprotein cholesterol
IDO1: indoleamine‐2,3‐dioxygenase1
IFN‐γ: interferon‐γ
IL‐1β: interleukin‐1β
IL‐6: interleukin‐6
Kyn: Kynurenine
LDL‐C: low‐density lipoprotein cholesterol
LPS: lipopolysaccharide
PLT: platelet
RBC: red blood cell
RNAseq: RNA‐sequencing
rRNA: ribosomal RNA
SD: standard deviation
T‐bil: total bilirubin
T‐cho: total cholesterol
TG: triglyceride
TNF‐α: tumor necrosis factor‐α
TP: total protein
Trp: Tryptophan
UA: uric acid
WBC: white blood cell

GPR142 is a G‐protein‐coupled receptor belonging to class A, rhodopsin family. It was recently de‐orphanized as an aromatic amino acid receptor [1]. Among aromatic amino acids, tryptophan is the most potent agonist for the receptor, followed by phenylalanine [1, 2, 3]. The highest expression level of GPR142 is observed in the islet of the pancreas [2, 4, 5, 6], but moderate levels of expression have also been seen in the gastrointestinal tract in rodents including, in the stomach, the duodenum, the jejunum, the ileum and the colon [2, 3, 4]. Stimulation of GPR142 in rodents by either tryptophan or synthetic agonist stimulates glucose‐induced insulin secretion [1, 2, 3, 4, 7], and other gastrointestinal hormones including glucagon [3], GIP [2, 3], GLP‐1 [2, 3] and CCK [3]. In humans, GPR142 is also highly expressed in the pancreatic islets [2, 7], and stomach, and tryptophan and GPR142 agonist enhance glucose‐stimulated insulin secretion from isolated human islets [2, 3].

Recently, Murakoshi et al. reported that GPR142 knockout mice showed resistance to collagen antibody‐induced arthritis (CAIA) and that TNF‐α and IL‐1β production induced by LPS were significantly attenuated in GPR142 knockout mice [8]. Furthermore, a GPR142 antagonist, CLP‐3094, significantly improved arthritis in CAIA model mice. These results suggest that GPR142 may have roles in the regulation of inflammation and the possible link between GPR142 and proinflammatory cytokines.

We previously found that GPR142 is highly expressed in the ghrelin‐producing cell line MGN3‐1 cells [9]. Tryptophan strongly stimulates ghrelin secretion from MGN3‐1 cells or primary‐cultured gastric epithelial cells *in vitro* through GPR142 [9]. Furthermore, proinflammatory cytokines directly suppressed ghrelin expression in MGN3‐1 cells [10]. Given that MGN3‐1 cells retain both GPR142 and proinflammatory cytokine pathways, they are considered to be a useful tool for dissecting the GPR142 and cytokine signaling interactions.

In this study, in order to understand the GPR142 and cytokine signaling interactions, we examined the relationship between proinflammatory cytokines and GPR142 expression by using cellular, animal models, and human stomach samples obtained from morbidly obese patients who underwent sleeve gastrectomy.

## Subjects, materials, and methods

### Cell culture and batch incubation studies

MGN3‐1 cells were previously established in our laboratory [11], αTC cells were purchased from ATCC (ATCC CRL‐2934), and MIN6 was kindly provided by Dr. Miyazaki. MGN3‐1 cells were cultured as previously described [11]. MGN3‐1 cells, αTC cells, and MIN6 cells were seeded at 5 × 10^5^ cells·well^−1^ and cultured for 24 h in 12‐well plates. Cells were incubated with 0.5, 5, and 50 ng·mL^−1^ TNF‐α (Wako, Osaka, Japan), 0.5, 5, and 50 ng·mL^−1^ IL‐6 (BioLegend, San Diego, CA, USA), and 0.05, 0.5, and 5 pg·mL^−1^ IL‐1β (BioLegend, San Diego, CA, USA).

### Animal study

We used male C57/BL6 mice (Japan SLC, Inc., Shizuoka, Japan). Animals were maintained on a 12‐h light/12‐h dark cycle and fed a standard diet (DC‐8, 343 kcal/100 g; Japan CLEA, Tokyo, Japan). Eight‐week‐old mice with essentially the same body weight were intraperitoneally injected with 0.5 g·kg^−1^ body weight of lipopolysaccharide (LPS; Sigma‐Aldrich, St.Louis, MO, USA) dissolved in saline, or with saline only (*n* = 10, each, as determined by preliminary experiments) for 3 consecutive days, without randomization and blinding. Body weights and plasma ghrelin levels were determined at day 4. Stomach samples were obtained at day 4 after cervical dislocation, and TNF‐α, GPR142, IFN‐γ, IDO1, and ghrelin mRNA levels were evaluated. All experimental procedures were approved by the Wakayama Medical University Committee on Animal Research; No. 1002. All animals' data were included in this study.

### Patients, sample collection

Enrolled in this study were eighteen morbidly obese patients who underwent laparoscopic sleeve gastrectomy in the Wakayama Medical University Hospital between 2018 and 2022 (Table 1). Body composition was measured with impedance analyzer (InBody 770, Biospace Co., Ltd, Seoul, South Korea). Routine preoperative lab tests including complete blood cell counts, serum chemistry, hormone assays, and anthropometric measurement were conducted 1–14 days before the operation. Tissue samples from three regions (fundus, body, and antrum) of the resected stomach were collected, immediately frozen, and stored at −80°C. This study protocol was conducted according to the guidelines set forth in the Declaration of Helsinki and all procedures involving human subjects/patients were approved by the Wakayama Medical University Hospital Ethics Committee; No.1862. Written informed consent was obtained from all patients.

**Table 1:** Anthropometric measurements before surgery.

Patient no.		1	2	3	4	5	6	7	8	9
Sex		F	M	M	F	M	F	F	M	M
Age	(y.o.)	41	51	53	66	52	32	52	45	30
Height	(cm)	167	166	178	155	170	148	156	169	165.5
Weight	(kg)	146.4	93.4	137.6	95.4	104.0	92.7	80.9	106.7	115.0
BMI	(kg·m^−2^)	52.5	33.9	43.4	39.7	36.0	42.3	33.2	37.4	42.0
Skeletal muscle	(kg)	37.6	51.6	75.4	43.8	55.0	40.5	37.1	56.6	61.5
Body fat	(kg)	79.6	38.8	57.8	49.3	45.8	49.9	41.6	46.7	50.1
%body fat	(%)	54.4	41.6	42.0	51.7	44.0	53.8	51.4	43.7	43.6

Patient no.		10	11	12	13	14	15	16	17	18
Sex		M	M	M	M	F	M	F	M	F
Age	(y.o.)	38	30	28	45	54	58	30	59	48
Height	(cm)	178	167	174	164	160	174	155	162	155
Weight	(kg)	186.1	99.8	131.2	160.2	126.9	107.7	95.4	123.3	86.6
BMI	(kg·m^−2^)	58.7	35.8	43.3	59.6	49.6	35.6	39.7	47.0	36.0
Skeletal muscle	(kg)	87.1	53.3	71.4	73.8	32.0	35.2	27.7	30.8	44.3
Body fat	(kg)	94.1	43.2	55.5	82.6	70.5	44.3	45.6	67.1	39.7
%body fat	(%)	50.5	43.3	42.3	51.6	55.5	41.2	47.9	54.4	45.8

### Primary culture of human gastric epithelium

Human gastric mucosal epithelium from the resected stomach, measuring 6–9 cm^2^ were washed twice with PBS and then digested in a solution containing 1.5 mg·mL^−1^ of Type I collagenase and 0.5 mg·mL^−1^ of dispase in DMEM supplemented with 10% FBS at 37 °C for 60 min. The gastric mucosal epithelium was then gently scraped off using a spatula. The cells were collected and counted after being passed through a cell strainer. Obtained cells were seeded at a density of 1 × 10^6^ cells per well in a 12‐well plate, and cytokine addition was initiated approximately 3 h after sample collection. Cells were incubated with 100 ng·mL^−1^ TNF‐α (Wako, Osaka, Japan) and 100 ng·mL^−1^ INF‐γ (Abcam, Cambridge, UK) for 20 h.

### Measurements of plasma ghrelin, tryptophan and kynurenine

Plasma ghrelin concentration was determined by AIA‐600II (Tosoh, Tokyo, Japan) as previously described [12]. Plasma Tryptophan and Kynurenine concentration were determined by Kynurenine/Tryptophan ratio ELISA pack (ImmuSmol, Bordeaux, France) following the manufacturer's protocol.

### RT‐PCR and real‐time quantitative RT‐PCR

RT‐PCR and real‐time quantitative RT‐PCR was conducted as reported [11] using the primers in Table S1 with Power SybrGreen. Human cytokines and 18S rRNA mRNAs were quantitated by the following ABI reagents: Human TNF, Hs00174128_m1, Human IL‐6, Hs00985639_m1, Human IL‐1β, Hs01555410_m1, Human 18S, Hs99999901_s1. Data were normalized to 18S rRNA.

### Public data sets analysis

An RNAseq data set of human stomach biopsy samples, deposited by Dr. Thorell et al. (Accession number: E‐MTAB‐3689), which include patients with *H. pylori* infection at various stages of gastritis as well as *H. pylori* negative controls, was downloaded from Array Express (https://www.ebi.ac.uk/arrayexpress/). The obtained FASTQ files were mapped to the human genome by hisat2 (http://daehwankimlab.github.io/hisat2/). Gene expression levels were quantified using StringTie (https://ccb.jhu.edu/software/stringtie/). The calculated FPKM values were used for correlation analysis.

### Statistical analysis

Inter‐group comparisons were made by ANOVA, and correlation among variables was calculated using the Pearson's correlation coefficient. Differences of *P* < 0.05 were considered to be significant. Statistical analyses were performed using JMP (SAS, Cary, NC, USA).

## Results

**Fig. 1:**
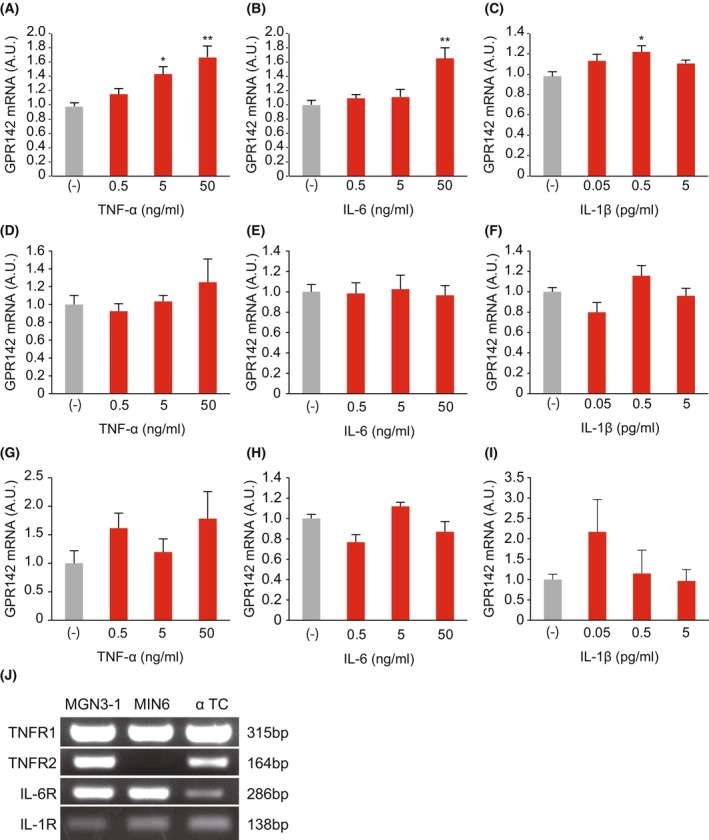
Effects of proinflammatory cytokines on the expression level of GPR142 mRNA in MGN3‐1, MIN6, and αTC cells. GPR142 mRNA levels in MGN3‐1 cells (A–C), MIN6 cells (D–F), and αTC cells (G–I), at 24 h after the addition of TNF‐α (A, D, G), IL‐6 (B, E, H), and IL‐1β (C, F, I). (J) TNF receptor 1 (TNFR1), TNF receptor 2 (TNFR2), IL‐6 receptor (IL‐6R), and IL‐1 receptor (IL‐1R) expression in MGN3‐1, MIN6, and αTC cells. AU, arbitrary units, *n* = 5–6. The results are presented as the mean ± SEM. *P < 0.05, **P < 0.01 by ANOVA and *post‐hoc* test.

To see if proinflammatory cytokines directly modulate GPR142 expression, we added TNF‐α, IL‐6, and IL‐1β to MGN3‐1 ghrelin‐producing cell line culture. We chose this cell line according to our previous finding that MGN3‐1 cells express relatively high levels of GPR142 mRNA [9] and the receptors for these cytokines [10]. We found that addition of TNF‐α, IL‐6, and IL‐1β significantly increased GPR142 mRNA expression in MGN3‐1 cells (Fig. 1A–C). We did not find any changes in GPR142 mRNA levels after addition of these cytokines to other known GPR142‐expressing cells, pancreatic β cell line, MIN6 cells (Fig. 1D–F) or pancreatic α cell line, αTC cells (Fig. 1G–I), despite the expression of the receptors for these cytokines (Fig. 1J). Given these findings, we decided to focus on the regulation of GPR142 by cytokines in the stomach.

**Fig. 2:**
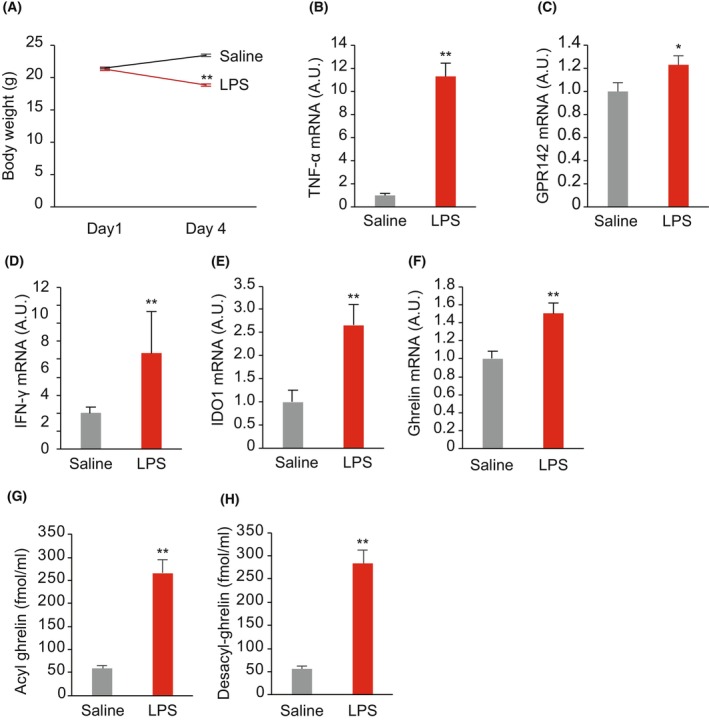
GPR142 mRNA expression levels in the stomach of mice 3 days after LPS injection. (A) Body weights of mice, who were injected with LPS from day 1 to day 3. (B–F) mRNA expression levels of TNF‐α (B), GPR142 (C), IFN‐γ (D), IDO1 (E), and ghrelin (F). (G, H) Plasma ghrelin (G) and desacyl‐ghrelin (H) levels of mice at day 4. A.U., arbitrary units, *n* = 10. The results are presented as the mean ± SEM. *P < 0.05, **P < 0.01 by Student's *t*‐test.

We examined if inflammation could stimulate GPR142 expression in the stomach of mice injected with LPS. Once daily injection of LPS to mice for 3 days significantly decreased the body weights of mice (Fig. 2A) and elevated TNF‐α expression in the stomach (Fig. 2B), confirming the induction of inflammation in the stomach. The expression of GPR142 was significantly elevated in the mouse stomach (Fig. 2C), which was in accordance with the observation of *in vitro* experiments. IFN‐γ (Fig. 2D) and IDO1 (Fig. 2E) mRNA levels were also increased. As for ghrelin, the mRNA levels in the stomach and plasma ghrelin levels were all increased (Fig. 2F–H).

**Fig. 3:**
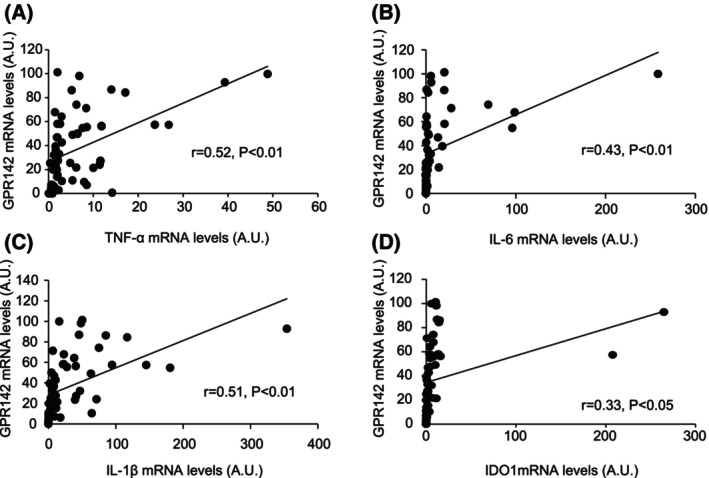
The correlation between GPR142 and TNF‐α, IL‐6, IL‐1β, and IDO1 mRNA levels in the stomach samples of human morbidly obese patients. The correlation between GPR142 and TNF‐α (A), IL‐6 (B), IL‐1β (C), or IDO1 (D) mRNA levels in the stomach samples obtained from morbidly obese patients who underwent sleeve gastrectomy. The mRNA expression levels were measured at three positions on each stomach samples.

We next examined if there are any relationship between GPR142 and proinflammatory cytokine expressions in human stomach samples. We recruited 18 morbidly obese patients (Table 1: 7 females, 11 males; mean age ± SD: 45.1 ± 11.3 years old; mean BMI ± SD:42.5 ± 8.0 kg·m^−2^), each of whom underwent sleeve gastrectomy in our university hospital. We examined the relationship between GPR142 mRNA expression levels with the mRNA expression levels of proinflammatory cytokines including TNF‐α, IL‐6, and IL‐1β in the stomach. The GPR142 mRNA levels were positively correlated with TNF‐α (*r* = 0.50, *P* < 0.01; Fig. 3A), IL‐6 (*r* = 0.43, *P* < 0.01; Fig. 3B), IL‐1β (*r* = 0.51, *P* < 0.01; Fig. 3C), and with IDO1 (*r* = 0.33, *P* < 0.05, Fig. 3D) mRNA levels in the stomach samples. We analyzed the relationships between GPR142 mRNA expression levels in the stomach samples and the anthropometric measurements (Table 1) and lab test values obtained before the surgery, which include complete blood cell counts, AST, ALT, total protein, albumin, total bilirubin, total cholesterol, triglyceride, HDL‐C, LDL‐C, uric acid (UA), creatinine, eGFR, CRP, glucose, HbA1c, c‐peptide, insulin, plasma acyl ghrelin, and desacyl‐ghrelin (Table 2). We found negative correlation between the expression levels of GPR142 mRNA in the stomach with LDL‐C (*r* = −0.55, *P* = 0.01). No other correlation was observed GPR142 mRNA levels and lab test values including plasma ghrelin levels. We found weak negative correlation between the expression levels of GPR142 mRNA in the stomach with serum kynurenine/tryptophan ratio, although it did not reach statistical significance (*r* = 0.38, *P* = 0.12).

**Table 2:** Lab test values before the surgery.

Patient no.		1	2	3	4	5	6	7	8	9	10	11	12	13	14	15	16	17	18
WBC	(×10^2^ /μL)	67.1	65.7	62.9	74.2	55.9	45.8	53.8	83.7	69.8	62.3	62.3	121.6	78.3	69.1	63.7	99.8	73.5	50.0
RBC	(×10^4^ /μL)	479	486	428	491	476	451	428	521	493	506	610	610	470	518	538	515	543	433
PLT	(×10^4^ /μL)	28.9	20.8	22.1	23.6	20.2	22.6	17.4	23.2	33.8	24.2	19.2	33.2	28.2	33.2	19.2	26.3	25.4	27.1
AST	(IU·L^−1^)	15	36	24	26	35	19	37	46	21	35	31	42	17	121	28	23	37	26
ALT	(IU·L^−1^)	11	78	31	19	76	12	41	65	29	66	50	66	12	136	47	45	99	44
TP	(mg·dL^−1^)	7.5	6.7	7.3	6.7	6.0	6.7	6.7	7.2	7.3	7.1	8.4	7.6	7.4	7.5	7.3	7.0	7.0	6.8
Alb	(mg·dL^−1^)	3.9	4.1	4.0	3.9	3.5	3.9	4.1	4.3	4.1	4.1	4.7	4.7	3.8	3.9	4.3	4.3	4.5	4.3
T‐bil	(mg·dL^−1^)	0.6	0.8	1.7	0.9	0.6	0.9	0.8	0.7	0.8	1.1	1.2	0.6	1.2	0.6	1.1	0.3	0.5	0.8
T‐Cho	(mg·dL^−1^)	202	137	146	202	134	185	214	177	247	237	177	203	192	161	168	177	166	181
TG	(mg·dL^−1^)	74	158	68	88	219	24	76	174	213	92	174	138	74	85	123	196	163	86
HDL‐C	(mg·dL^−1^)	47	38	40	65	44	40	63	48	35	51	65	30	42	41	37	33	38	50
LDL‐C	(mg·dL^−1^)	134	74	87	116	59	125	132	107	181	180	92	143	133	109	106	118	107	111
UA	(mg·dL^−1^)	6.0	4.8	7.0	5.2	9.2	6.7	7.3	6.4	6.5	7.6	8.0	5.7	5.7	10.5	4.5	6.9	5.3	4.7
Cre	(mg·dL^−1^)	0.64	0.97	0.81	0.64	1.00	0.63	0.53	1.09	0.78	0.68	0.78	0.72	0.82	0.87	1.09	0.58	0.74	0.64
eGFR	(mL/min/1.73m^2^)	80.5	65.3	78.6	70.2	62.4	87.9	92.4	59.2	95.9	103.4	95.9	106.8	80.8	53.4	55.0	99.0	83.7	76.9
CRP	(mg·dL^−1^)	0.23	0.02	0.10	0.17	0.82	0.09	0.28	0.09	0.59	0.10	0.28	1.10	0.52	1.00	0.13	0.30	0.14	0.12
Glucose	(mg·dL^−1^)	92	142	103	122	108	72	100	132	90	88	163	134	85	109	99	155	132	101
HbA1c	(%)	5.2	7.5	5.1	7.2	6.3	4.9	5.6	7.1	5.7	5.0	8.8	7.9	5.1	6.2	5.5	8.2	6.5	5.7
CPR	(ng·mL^−1^)	2.40	3.53	2.35	2.53	5.32	1.86	2.96	5.88	4.11	ND	3.43	5.47	2.41	6.91	3.14	5.24	8.27	2.74
Insulin	(μU·mL^−1^)	8.2	10.5	10.1	9.5	16.4	6.3	16.5	26.8	19.5	23.1	ND	ND	12.4	47.8	11.9	20.9	33.1	15.5
Acyl Ghrelin	(fmol·mL^−1^)	6.49	3.18	4.22	12.49	8.46	13.64	3.53	9.52	5.11	2.13	3.17	25.06	4.81	6.58	10.75	27.13	4.06	4.03
Desacyl‐Ghrelin	(fmol·mL^−1^)	19.21	19.86	10.15	33.2	15.46	40.81	31.79	38.65	23.06	8.20	8.56	119.22	8.64	37.00	24.57	40.71	24.85	9.17
Trp	(μmol·L^−1^)	36.5	46.1	72.4	43.4	33.2	25.2	39.2	40.3	34.7	34.0	70.1	56.0	29.5	46.8	23.3	27.8	33.9	32.3
Kyn	(nmol·L^−1^)	1644.3	2104.0	1787.5	2722.8	2141.7	1194.9	1441.4	2356.9	1812.7	1640.7	2405.9	1796.6	2806.4	3593.0	2162.9	1681.2	1571.6	2557.7

**Fig. 4:**
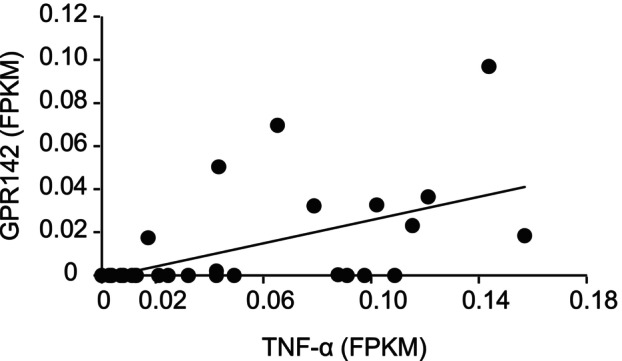
The correlation between GPR142 and TNF‐α FPKM values in the RNAseq dataset of stomach biopsy samples with *H. pylori* infection. The FPKM values of GPR142 and TNF‐α in the publicly available RNAseq dataset of stomach human biopsy samples with *H. pylori* infection (Array Express: E‐MTAB‐3689).

Next, we examined the GPR142 and cytokines expression levels in the publicly available RNAseq datasets of stomach samples to see if these correlations were also observed in the stomach samples with inflammation of another cohort. We analyzed a dataset of human stomach biopsy samples with *H. pylori* infection deposited by Dr. Thorell et al. (Array Express: E‐MTAB‐3689). GPR142 FPKM values were significantly correlated with those of TNF‐α in the data set (*r* = 0.52, *P* < 0.01; Fig. 4), suggesting that the correlation is not specific to morbidly obese patient, although no significant correlations were observed between GPR142 and IL‐6, nor IL‐1β FPKM values (data not shown).

**Fig. 5:**
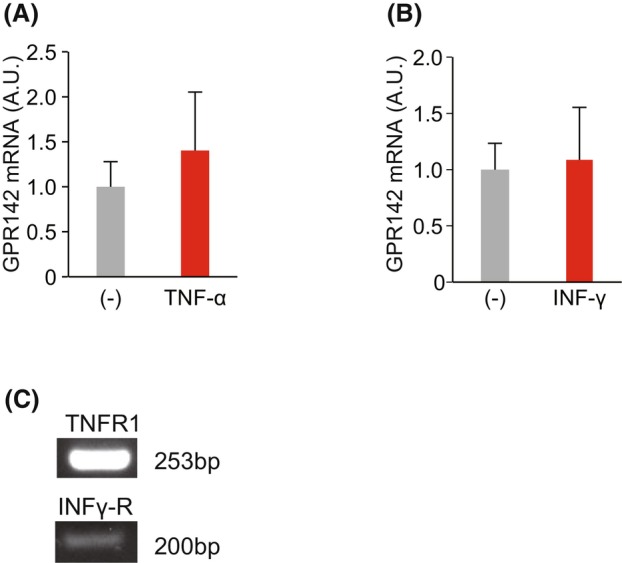
Effects of proinflammatory cytokines on the expression level of GPR142 mRNA in primary‐cultured human gastric epithelial cells. GPR142 mRNA levels in primary‐cultured human gastric epithelial cells at 20 h after the addition of TNF‐α (A) or INF‐γ (B). (C) TNF receptor 1 (TNFR1) and INF‐γ receptor (INF‐γR) expression in primary‐cultured human gastric epithelial cells. A.U., arbitrary units, *n* = 6. The results are presented as the mean ± SEM.

Finally, we used primary‐cultured human gastric epithelial cells to better replicate the environment of the human body. Primary‐cultured human gastric epithelial cells were incubated with TNF‐α or IFN‐γ for 20 h, but no significant changes in GPR142 mRNA expression levels were observed (Fig. 5), despite the expression of the receptors for these cytokines.

## Discussion

In this study, we found that TNF‐α, IL‐6, and IL‐1β directly upregulate GPR142 in the ghrelin‐producing cells. The elevation of GPR142 mRNA levels in the stomach was also observed *in vivo* mouse model with LPS injection. In addition, GPR142 mRNA expression levels in stomach samples of morbidly obese patients were positively correlated with stomach TNF‐α, and IL‐6 mRNA levels. The correlation between GPR142 and TNF‐α expression levels was also observed in the stomach biopsy samples of *H. Pylori*‐infected patients. These results suggest that GPR142 expression levels are under the direct control of cytokine signaling. As far as we know, this is the first report to reveal the regulatory factors for GPR142 levels. Contrary to predictions, we failed to demonstrate the direct regulation of GPR142 mRNA levels by TNF‐α in human primary‐cultured gastric epithelial cells. This may be caused by the heterogeneous nature of the primary‐cultured gastric epithelial cells or by cellular damage during the preparation of the culture. Previous report indicates that the expression GPR142 is primarily limited to endocrine cells, including at least ghrelin‐producing cells, in the stomach. The primary‐cultured human gastric epithelial cells used in our study contained various other cell types, which may influence the results. Additionally, because we obtained the stomach samples during surgery, there was a delay of several hours before conducting the experiment, which could have affected cellular responses.

The pathophysiological meaning of direct regulation of GPR142 levels by proinflammatory cytokines in the stomach is not yet clear. Considering that GPR142‐signaling enhances ghrelin secretion, the elevation of GPR142 expression by the cytokines may act as a counterbalance for suppression of ghrelin secretion during the inflammation [10]. Hataya et al. showed that ghrelin levels were suppressed after single LPS injection in rats [13]. In accordance with their findings, we previously confirmed that proinflammatory cytokines directly suppressed ghrelin production in ghrelin‐producing cells [10]. The suppressed ghrelin production during inflammation may be counterbalanced by the increased GPR142, whose activation stimulates ghrelin secretion [9].

Alternatively, the elevation of GPR142 can be regarded as a counter‐regulation for decreased ligands because serum tryptophan levels are decreased during inflammation due to increased metabolism through IDO1, which is strongly induced in the monocytes or macrophages [14]. Metabolites through this pathway, known as kynurenine pathway, has various biological actions including suppression of immune responses. Indeed, in this study, IDO1 mRNA levels were strongly elevated in the mice with LPS injection, and GPR142 levels were positively correlated with IDO1 mRNA levels in the stomach of morbid obesity patients. When we looked at the relationship between GPR142 mRNA expression and plasma tryptophan, however, we did not find significant correlation in the analysis of the data of our 18 patients. We observed a tendency for weak positive correlation between the expression levels of GPR142 mRNA in the stomach with serum kynurenine/tryptophan ratio, but it also did not reach statistical significance. This may be due to the small sample size or the study design, which only includes the stomach samples from morbidly obese patients who did not have severe inflammation. Although it is theoretically possible that kynurenine could elevate GPR142 expression levels, this appears unlikely based on the negative results from experiments with MGN3‐1 cells (Fig. S1). Further study will be needed to explore the role of GPR142 in the regulation of ghrelin levels during inflammation.

In summary, GPR142 mRNA expression levels were directly up‐regulated by TNF‐α, IL‐6 and IL‐1β in the ghrelin‐producing cell line and the levels were positively correlated with TNF‐α, IL‐6, IL‐1β and IDO1 mRNA levels in the human stomach samples. These results suggest that GPR142 expression is under the direct control of proinflammatory cytokines and may support further investigation of GPR142 roles in inflammation.

## Conflict of interest

The authors declare no conflict of interest.

### Peer review

The peer review history for this article is available at https://www.webofscience.com/api/gateway/wos/peer‐review/10.1002/2211‐5463.13973.

## Author contributions

YU, HIw, and TA designed the work. YU, HIw, TE, MB, AD, NM, SM, HIn, HA, NF, KH, TO, MN, HY, and TA collected and analyzed data. YU, HIw, SM, HIn, HA, NF, KH, TO, MN, TM, HY, and TA interpreted data. YU, HIw, and TA drafted the manuscript. All authors reviewed the manuscript.

## Supporting information


**Fig. S1.** Effects of L‐kynurenine on the expression level of GPR142 mRNA in MGN3‐1 cells.
**Table S1.** Primers.
