# Supplementary material for: Tryptophan‐sensing receptor GPR142 expression levels are directly regulated by proinflammatory cytokines in ghrelin‐producing cells
Source: FEBS Open Bio. 2025 Jan 30;15(5):763–72. doi: 10.1002/2211-5463.13973 (PMC12051013; doi:10.1002/2211-5463.13973)
Supplement: Supplementary file 1 — Fig. S1. Effects of L‐kynurenine on the expression level of GPR142 mRNA in MGN3‐1 cells. Table S1. Primers. [file FEB4-15-763-s001.pdf]

Supplementary figure legends

Supplementary figure 1. Effects of L-kynurenine on the expression level of GPR142 mRNA in MGN3-1cells

GPR142 mRNA levels in MGN3-1 cells at 24 h after the addition of L-kynurenine. A.U.: Arbitrary units, n=6. The results are presented as the mean ± SEM.

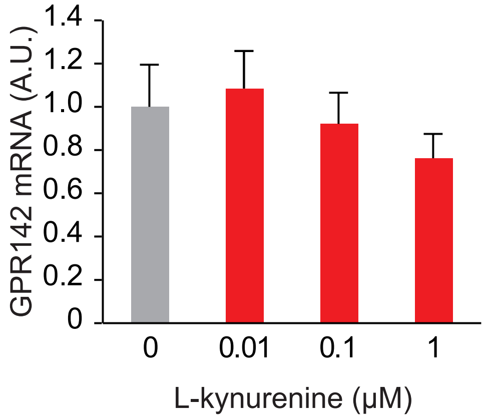
GPR142 mRNA (A.U.)
1.4
1.2
1.0
0.8
0.6
0.4
0.2
0
0
0.01
0.1
1
L-kynurenine (μM)

Supplemental Table 1. Primers

mouse TNFR1	sense	5'-ACCGTGACAATCCCCTGTAA-3'
	antisense	5'-CTCTTTGACAGGCACGGGAT-3'
mouse TNFR2	sense	5'-TGCTGTGCTAAGTGTCCTCCT-3'
	antisense	5'-TTCCATTTGGGGCTCTTGAAC-3'
mouse IL-6R	sense	5'-CTGGGTGTACTCTGGCTCAC-3'
	antisense	5'-TCCCGTTGGTGGTGTTGATTT-3'
mouse IL-1R1	sense	5'-AACCCAAGAGGCTCTGACAC-3'
	antisense	5'-CGAGACAACGTTTGAGTTTGCT-3'
human TNFR1	sense	5'-ATTGGACTGGTCCCTCACCT-3'
	antisense	5'-ACCTGACCCATTTCCTTTCGG-3'
human INFγR1	sense	5'-TTTCTCCTACCCCTTGTCATGC-3'
	antisense	5'-ATTAGTTGGTGTAGGCACTCC-3'
